# Two-dimensional tracking and TDI are consistent methods for evaluating myocardial longitudinal peak strain in left and right ventricle basal segments in athletes

**DOI:** 10.1186/1476-7120-5-7

**Published:** 2007-02-07

**Authors:** Laura Stefani, Loira Toncelli, Marco Gianassi, Paolo Manetti, Valentina Di Tante, Maria Robertina Concetta Vono, Andrea Moretti, Brunello Cappelli, Gianni Pedrizzetti, Giorgio Galanti

**Affiliations:** 1 Non-Invasive Cardiac Laboratory of Sports Medicine Unit – University of Florence, Italy; 2 Dept. Civil and Environmental Engineering, University of Trieste, Italy

## Abstract

**Background:**

Myocardial contractility can be investigated using longitudinal peak strain. It can be calculated using the Doppler-derived TDI method and the non-Doppler method based on tissue tracking on B-mode images. Both are validated and show good reproducibility, but no comparative analysis of their results has yet been conducted. This study analyzes the results obtained from the basal segments of the ventricular chambers in a group of athletes.

**Methods:**

30 regularly-trained athletes were submitted to an echocardiography at rest and after handgrip. Starting from the four-chamber view, overall myocardial function and regional velocities were evaluated. The images obtained were processed to determine strain in left and right ventricle basal segments. Strain was calculated using the TDI method and a validated "speckle tracking" or, more correctly, "feature tracking" algorithm. The statistical analysis included a Student's t-test (p < 0.05).

**Results:**

The range of strain values obtained is in agreement with the data reported in the literature. In the left ventricle (LV) the average strain values of the basal segments calculated with TDI on IVS and LW at rest and after stress were: -21.05 ± 3.31; -20.41 ± 2.99 and -20.05 ± 2.61; -21.20 ± 2.37, respectively. In the right ventricle (RV) the same method gave IVS and LW strain values at rest of -22.22 ± 2.58 ; -24.42 ± 5.84, and after HG of -22.02 ± 5.20 ;-23.93 ± 6.34. The values obtained using feature tracking were: LV at rest -20.48 ± 2.65 for IVS, and -21.25 ± 2.85 for LW; LV after HG: -19.48 ± 3 for IVS and -21.69 ± 3.85 for LW. In RV at rest: -21.46 ± 3.25 for IVS and -24.13 ± 5.86 for LW; RV after HG: -24.79 ± 7.9 for IVS and -24.13 ± 7.0 for LW. Tissue Doppler and "feature tracking" methods showed the respective consistency of the results in the basal segments of myocardial ventricle walls.

**Conclusion:**

Provided that echographic imaging is good, strain can be computed in athletes by both Doppler-derived and tracking methods. It is technically feasible to use both -interchangeably, at least in basal segments.

## Background

Conventional strain (*S*), a measure of regional deformation, can be calculated from the gradient of velocity from Tissue Doppler Imaging [1] or by "feature tracking" techniques performed on grey scale (B-mode) images. "Feature tracking" is a more appropriate term than "speckle tracking" because these techniques actually allow assessment of myocardial deformation also in the absence of speckles [2,3]. Mirsky and Parmely defined "strain" as a dimensionless quantity that represents the percentage change in dimension from a rest state to one achieved after application of a force (stress) [4], and particularly myocardial longitudinal strain is negative in the shortening and positive in the lengthening of a myocardial segment from its reference "R" state.

Both methods provide reproducible results [5-7], depending mostly on the high quality of the images and experience of the operator, but they present different strengths and limitations. The TDI technique quantifies only the axial components of velocity, along the direction of the ultrasound wave, and the angle dependence reduces its accuracy when applied near the myocardial apex where the tissue is typically not aligned with the ultrasound beam. On the other hand, "feature tracking" has the weaknesses of poor resolution of the lateral myocardial wall; in fact, in the grey scale images, interferences from backscattered ultrasound from neighboring structures produce a random speckle pattern, and the low frame rate of grey scale images may lead to under-sampling. Recently, a new scanning technology has been proposed which simultaneously acquires not only high-quality 2-dimensional images with an adequate frame rate for grey scale imaging, but also high frame rate tissue Doppler data [8,9]. However, this automated analysis method cannot analyze as many segments as can be done manually [10], and the combined use of both methods is still not a viable solution. There is no direct evidence in literature of the consistency of the two non-automated methods, at least in basal segments where angle dependence is not influential, thus ruling out the possibility of employing the two methods interchangeably.

This study aims at evaluating the uniformity of the results on longitudinal peak strain values obtained with feature tracking and Doppler-derived methods in left and right ventricle basal segments, in a group of athletes at rest and after an isometric stress test. To overcome the limitations, only the high-quality images were considered, and only basal segments of the left and right ventricles were investigated. The stress test was included in the study protocol to enhance the differences in myocardial deformation.

## Methods

### Design

30 soccer-players, 25 ± 3 years, regularly trained three times a week for 10 months a year for almost five years, were studied (Table 1). The athletes were submitted to a complete clinical evaluation which included anamnesis and an objective examination to exclude important lung, heart, or metabolic diseases. A complete cardiological check-up including basal ECG was performed. The study protocol was approved by the ethics committee of the University of Florence and subjects gave their written informed consent

**Table 1:** Baseline characteristics of the study population

**Athletes **	Age (years)	Gender (M/F)	Height (cm)	Weight (Kg)
30	25 ± 3	13/2	178 ± 12	71 ± 13

The table shows the main characteristics of the group of athletes studied

Following the study protocol, each subject remained at rest for 10 minutes, then was submitted to a trans-thoracic echographic test at rest and after a hand-grip stress (HG). The latter was performed with an adequately-calibrated handle dynamometer, placed in the athlete's dominant hand in line with his forearm and hanging straight down his side. The athletes were required to keep the handle pressed at 30% of the maximal effort previously established for a period of 3 minutes Maximum grip strength without swinging of the arm was then determined. Systolic and diastolic blood pressure were measured at rest and at the end of maximal stress. This sequence was performed twice for each athlete to record the 2D images, which were processed by both TDI and B-mode tracking methods to calculate strain values. The Ejection Fraction was calculated at rest and after HG from the apical 4-chamber view using Simpson's algorithm. The study protocol was repeated twice 7 days later by a different, expert operator.

### Echocardiographic recordings and image acquisition

Echocardiographic imaging was performed with subjects lying in left lateral position. Echo GE Vivid 7 and Philips SONOS 5500 echocardiographs were utilized to record the images of the two ventricles. In accordance with the recommendations of the American Society of Echocardiography [11], the usual parameters were measured in M-mode, two-dimensional mode, and continuous and pulsed-wave Doppler echocardiography. LV (Left Ventricular) 4-chamber-view images were obtained with conventional 2D grey scale echocardiography. Two-dimensional grey scale images with real-time left and right ventricular endocardial detection were stored digitally for subsequent offline analysis. In the TDI method, ellipsoid ROI was defined so that the left and right endocardial layer could be encapsulated for the whole cardiac cycle. The feasibility of feature-tracking analysis was ensured by images free of reverberation and with a good lateral wall resolution.

### Analysis of deformation of left and right ventricle basal segments

With the Vivid 7 echocardiograph, it was possible to determine LPS (longitudinal peak strain) in real time with the Tissue Doppler method. With the Philips SONOS 5500, the myocardial images were collected for the next feature tracking analysis. Tracking and subsequent strain calculations were performed in MatLab (The MathWorks, Natick MA, U.S.A.) with custom software based on a previously-validated algorithm [5,7].

**Figure 1:**
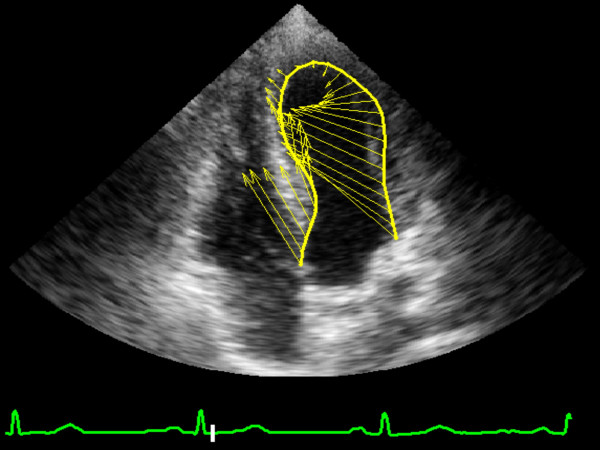
Image of "feature tracking" calculation of systolic longitudinal strain in left ventricle at rest.

**Figure 2:**
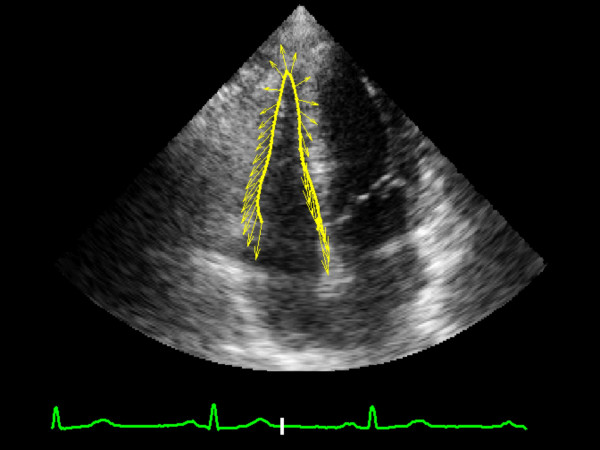
Image of "feature tracking" calculation of systolic longitudinal strain in right ventricle at rest.

**Figure 3:**
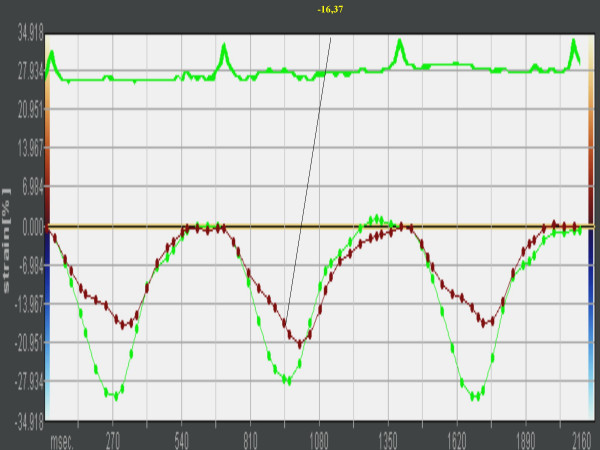
The curve shows a basal segment strain value by tissue tracking on B-mode clips.

**Figure 4:**
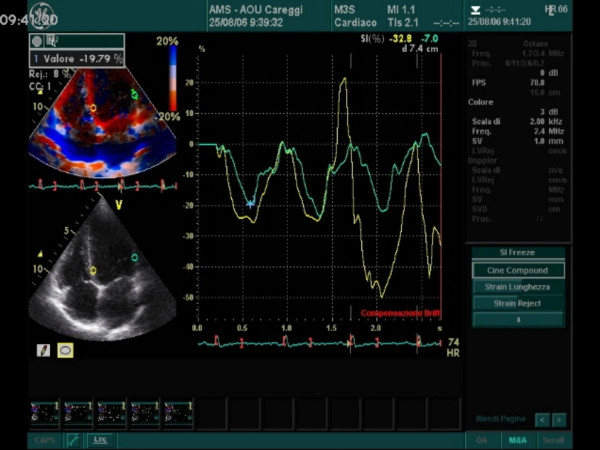
Systolic peak strain analysis at rest with Tissue Doppler : the panel shows left ventricle interventricular septum (IVS) and lateral wall (LW) values in basal segments.

**Figure 5:**
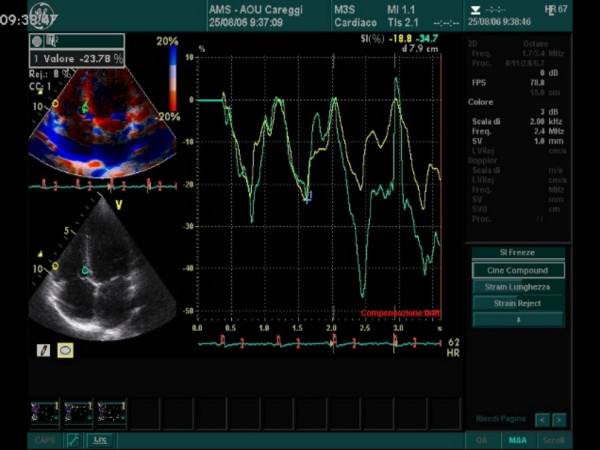
This panel shows right ventricle interventricular septum (IVS) and lateral wall (LW) systolic peak strain values with Tissue Doppler. All regions are located in basal segments.

Longitudinal Peak Strain was calculated in the lateral wall (LW) and interventricular septum (IVS) basal segments of the left and right ventricles of the athlete's heart (Figures 1, 2, 3, 4, 5). In order to reduce regional artifacts, only the high-quality images without reverberations were considered and processed. A good echocardiographic myocardial view was always obtained in the athletes enrolled in this study, allowing identification of the endocardial border and left and right ventricle wall thickening.

Following the indications in the literature [12,13], and in order to reduce random noise, each sample was obtained by averaging more than one consecutive heart cycle (usually three), setting the frame rate between 75–80 FPS (Frames Per Second). These settings are recommended to combine temporal resolution with an acceptable lateral definition, to enhance the feasibility of the tracking technique, and to decrease the possibility of angle problems in the Doppler-derived method. With the TDI technique, for each segment a stationary region of interest (ROI) was manually set in early systole in the middle wall portion of each segment. The ROI size was 5 mm laterally and it was adjusted longitudinally to cover the length (mean 20 mm) of the segment. This procedure was performed in double-blind by two expert operators a week later and following the same protocol. All the echocardiographic analyses were performed without knowledge of the results of the reference methods. To assess inter-observer variability, 6 echocardiographic tests were randomly selected and then independently analyzed by two different observers with the two methods.

### Statistical analysis

Values were expressed as [average ± SD]. Comparisons among data were performed with Student's t test, p < 0.05. For intra-observer and inter-observer variability 95% limits of agreement were used.

## Results

### Feasibility of echocardiographic data

In all the athletes enrolled, the myocardial wall basal segments were analyzed using the two methods, and only the good-quality echocardiographic images were processed. One reason for exclusion was inability to obtain the ROI (region of interest) closely enough to the shape of the left and right ventricular endocardium, which would result in underestimation of data and their contamination by signal components outside the left ventricular cavity. The 4-chamber view was the one which allowed the best delineation of the ventricular cavity and the best detection of the endocardial border.

### Estimated global function and regional deformation

All the athletes maintained a normal EF % at rest and after stress, without any significant differences. The studied group showed a higher cardiac mass index than the upper limits of the normal range (132 ± 5 g/m^2^), confirming that the athletes were regularly trained. Heart rate and systolic blood pressure increased significantly after stress. The results of the echocardiographic parameters are reported in Table 2

**Table 2:** Clinical and echocardiographic findings

	**Rest**	**Stress**	***p***
SBP mmHg	130 ± 5	145 ± 6	<0.05
DBP mmHg	75 ± 7	80 ± 9	ns
Heart rate	60 ± 7	73 ± 5	<0.05
LVEF %	58 ± 5 %	60 ± 3	ns
LA diam(mm)	35.12 ± 1.2	34 ± 2.3	ns
IVRT(ms)	81 ± 7.4	83.6 ± 6.38	ns
DTc(ms)	190.5 ± 13.4	178.5 ± 12.2	ns
E/A	1.4 ± 0.27	1.33 ± 0.9	ns

Legend. SBP: Systolic Blood Pressure; DBP: diastolic Blood Pressure; LVEF: Left ventricular Ejection Fraction; LA: Left Atrium; IVRT: IsoVolumetric Relaxation Time; DTc: Deceleration Time; E/A : E-wave A-wave peak velocity ratio.

The table shows the values of the main echocardiographic parameters considered in the study obtained at rest and after HG stress

The results of the strain analysis performed with the two different methods are shown in Table 3. The values refer to systolic peak strain on basal segments of the heart. Comparison of the values shows the consistency between the results of the Doppler-derived technique and those obtained with the feature tracking method. The values are also in agreement with the data reported in the literature [12-15]. The 95% limits of agreement of the two methods were: lower 0.51-upper 0.54 for TDI, and lower 0.44- upper 0.47 for tracking.

**Table 3:** Strain analysis: systolic longitudinal peak strain in basal segments.

**First session**				
	**LV REST**		**LV HG**	
	
	IVS	LW	IVS	LW
TDI	-21.05 ± 3.31	-20.41 ± 2.99	-20,05 ± 2.61	-21.20 ± 2.37
FT	-20.48 ± 2.65	-21.25 ± 2.85	-19.48 ± 3	-21.69 ± 3.85
*p*	Ns	ns	ns	ns
	
	**RV REST**		**RV HG**	
	
	IVS	LW	IVS	LW

TDI	-22.22 ± 2.58	-24.42 ± 5.84	-22.02 ± 5.20	-23.93 ± 6.34
FT	-21.46 ± 3.25	-24.13 ± 5.86	-24.79 ± 7.9	-24.13 ± 7.0
*p*	Ns	ns	ns	ns

**Second session, 7 days later**				

	**LV REST**		**LV HG**	
	
	IVS	LW	IVS	LW

TDI	-22.0 ± 2.40	-21.31 ± 3	-19,9 ± 2.70	-22.27 ± 3
FT	-21.32 ± 2.73	-20.44 ± 2.90	-20.62 ± 2.82	-20.80 ± 2.90
*p*	Ns	ns	ns	Ns

	**RV REST**		**RV HG**	
	
	IVS	LW	IVS	LW

TDI	-21.02 ± 2.80	-23.16 ± 4.34	-23.30 ± 5.72	-23.12 ± 5.23
FT	-20.52 ± 3.60	-22.14 ± 4.75	-24.62 ± 6.8	-24.19 ± 6
*p*	Ns	ns	ns	Ns

Legend. LV: Left Ventricle; RV: Right Ventricle; IVS: Inter Ventricular Septum; LW: Lateral Wall; TDI: Tissue Doppler Imaging method; FT: Feature Tracking method.

The table reports the strain values of the basal segments of the left and right ventricles obtained in two different sessions, with Tissue Doppler and feature tracking methods.

In addition, we wish to note that the longitudinal strain in the athlete's heart appears to be higher in the right than in the left ventricle, although the statistical differences are not sufficient to draw any conclusion.

## Discussion

Strain is considered a valid parameter for estimating myocardial contractility by deformation [2,3] and two main methods are currently available for its calculation: the standard Doppler-derived approach, and the newer one based on tissue tracking on B-mode clips. Both methods used by an expert operator show high reproducibility of the results [7-9], and new automated methods based on a combination of speckle tracking and tissue Doppler are now under development [10]. Both methods show good accuracy, but each presents different limitations that make comparisons difficult; for this reason an investigation of the uniformity of their results has not so far been conducted.

A careful comparison is feasible at least in interventricular septum (IVS) and lateral wall (LW) basal segments of left and right ventricles (LV, RV), where either method can be applied with equal accuracy, and the Doppler angle dependency does not interfere\souts with the TDI analysis. This study was conducted in a group of athletes that presented a high quality of the image where the tracking method was properly applied. Comparison was made of strain values as a major dimensionless measure of tissue contractility. The test was carried out both at rest and after handgrip stress to enhance variability and investigate the consistency of the results. It was found that Doppler-derived and "feature tracking" approaches show substantial agreement in the results, since no statistical differences were evident in the basal segments of either ventricle at rest and after HG effort. This study shows that, when high-quality images are recordable, the deformation of the basal heart segments at rest and after stress can be investigated by two different methods which lead to nearly the same results.

## Conclusion

### Possible applications and further development

The accurate identification of regional myocardial function in athletes by strain analysis is important for follow-up and management of the training itself. In this particular population, the physiological hypertrophy of the heart supports the hypothesis that longitudinal strain may present changes in basal segments that are nearly rectilinear. A mild increase in longitudinal strain values appears in the right more often than in the left ventricle, although a wider and heterogeneous group of athletes should be studied to investigate this particular trend. It should be remembered that the two methods present several limitations to a correct interpretation of the results that preclude routine use of the strain concept. The Doppler-derived method is limited by angle dependence, which makes it unable to evaluate LV deformation with uniform accuracy along the different ventricular walls and in the different echocardiographic projections. Feature tracking is performed on grey scale (B-mode) echocardiographic images where the local grey-scale pattern (including speckles) remains reasonably stable; unlike TDI, the velocity evaluated with this method is a vector, and does not therefore present any angle dependence. It can be applied with the same accuracy to the different myocardial regions; on the other hand it is a novel technique that requires further practical experience validation studies. Both methods are influenced by image quality, and in particular clinical conditions they can present limitations due to the physiological growth of the myocardial chambers, which prevents the perfect framing of the image in the echographic window.

In conclusion, strain values computed with the TDI-derived method and the tissue tracking technique are equivalent in the basal segment. The former has more literature to support it, but is limited to basal and low-median segments. Tracking appears to open up possibilities of extending strain analysis to the whole of the myocardial wall. It might allow complete analysis of the development of myocardial contractile adaptation in athletes in training and during their career.

The potential clinical applications of the two methods used to estimate myocardial contractility are not further explored here since this is not within the scope of the study and would necessarily imply the use of subjects with different pathologies.

## Competing interests

The author(s) declare that they have no competing interests.

## Authors' contributions

The initial idea for the study was of GG and LS and designed the study, and LS, GP, LT performed all the measurements and statistical analyses. LS wrote the manuscript and all the authors contributed to, read, and approved the final version.
